# αMSH prevents ROS-induced apoptosis by inhibiting Foxo1/mTORC2 in mice adipose tissue

**DOI:** 10.18632/oncotarget.16606

**Published:** 2017-03-27

**Authors:** Weina Cao, Meihang Li, Tianjiao Wu, Fei Feng, Tongying Feng, Yang Xu, Chao Sun

**Affiliations:** ^1^ College of Animal Science and Technology, Northwest A&F University, Yangling, Shaanxi 712100, China

**Keywords:** αMSH, Foxo1, ROS, apoptosis, mTOR

## Abstract

Alpha-melanocyte stimulating hormone (αMSH) is an important adenohypophysis polypeptide hormone that regulates body metabolic status. To date, it is well known that the disorder of hypothalamic αMSH secretion is related to many metabolic diseases, such as obesity and type II diabetes. However, the underlying mechanisms are poorly understood. In our study, we focused on the reactive oxygen species (ROS)-induced adipocyte apoptosis and tried to unveil the role of αMSH in this process and the signal pathway which αMSH acts through. Kunming white mice were used and induced to oxidative stress status by hydrogen peroxide (H_2_O_2_) injection and a significant reduction of αMSH were found in mice serum, while elevated ROS level and mRNA level of pro-apoptotic genes were observed in mice adipose tissue. What is more, when detect the function of αMSH in ROS-induced apoptosis, similar inhibitory trend was found with the oxidative stress inhibitor N-acetyl-L-cysteine (NAC) in ROS-induced adipocyte apoptosis and this trend is αMSH receptor *melanocortin 5 receptor* (*MC5R*) depended, while an opposite trend was found between αMSH and Foxo1, which is a known positive regulator of adipocyte apoptosis. Further, we found that the repress effect of αMSH in adipocytes apoptosis is acting through Foxo1/mTORC2 pathway. These findings indicate that, αMSH has a strong inhibitory effect on ROS-induced adipocyte apoptosis and underlying mechanism is interacting with key factors in mTOR signal pathway. Our study demonstrated a great role of αMSH in adipocyte apoptosis and brings a new therapeutic mean to the treatment of obesity and diabetes.

## INTRODUCTION

Alpha-melanocyte stimulating hormone (αMSH) is an endocrine hormone secreted by adenohypophysis. By binding to its receptors (MCRs), αMSH mediates multiple physiological functions, including metabolic regulation [1], neuroprotection [2], anti-inflammation [3] etc. In the function of metabolic regulation, αMSH can reduce food intake and increase energy consumption in peripheral tissues and organs via its receptors in the target tissues [4–5]. When αMSH-MC5R pathway is inhibited *in vivo*, mice show a great increase of lipid deposition and prone to develop obesity [6]. In mice adipocytes, adding of exogenous αMSH is found to promotes preadipocyte proliferation and enhance fatty acid oxidation [5, 7]. Reactive oxygen species (ROS), such as hydrogen peroxides, superoxide, hydroxyl radical etc., is a kind of oxygen containing chemical species that can promote oxidative stress. Low concentration of ROS is important in keeping redox balance and cell proliferation [8], while excessive ROS accumulation induces protein oxidation, lipid peroxidation and DNA damage, followed by cell death or apoptosis in PC-12 cell line and human colon cancer HCT116 cells [9–11]. Apoptosis plays a significant role in the maintenance of tissue homeostasis [12–13]. Taylor et al. find αMSH is a post-caspase suppressor of apoptosis in macrophages [14]. Intravitreal injection of an αMSH analog protects photoreceptor cells from death in a rat model of retinal dystrophy in a dose-dependent manner [15]. However, few literatures were found in the study of αMSH in adipocyte apoptosis and the regulatory mechanism in the process.

*Forkhead box class O 1* (*Foxo1*) is a Foxo family member that functions in adipocyte survival and apoptosis [16]. Foxos can increase cell death through intrinsic apoptotic pathway mediated by mitochondria [17]. In mammalian skeletal muscle, Foxo1 is shown having a inhibitory role in mTOR signaling [18]. In human lung cancer cell, acetylized *Foxo1* is also required for cell apoptosis and the depsipeptide-induced activation of *Bim* [19]. mTOR, as an evolutionary conserved protein complex negatively regulating catabolic pathways (autophagy and apoptosis) [20], is commonly used as the drug target in treatment to various types of cancer [21]. But, in adipocyte apoptosis, the relationship between Foxo1 and mTOR signal is still not well studied.

In this study, we explored the role of αMSH in ROS-induced apoptosis in mice adipose tissue. We also investigated the role of *Foxo1* and mTORC2 signal in the process of αMSH inhibiting adipocytes apoptosis. This work aimed to elucidate a novel function of αMSH in the regulation of cellular oxidative stress and apoptosis, implying the potential of αMSH as a drug for treating metabolism syndrome.

## RESULTS

### αMSH inhibited H_2_O_2_-induced oxidative stress and apoptosis in adipose tissue

**Figure 1:**
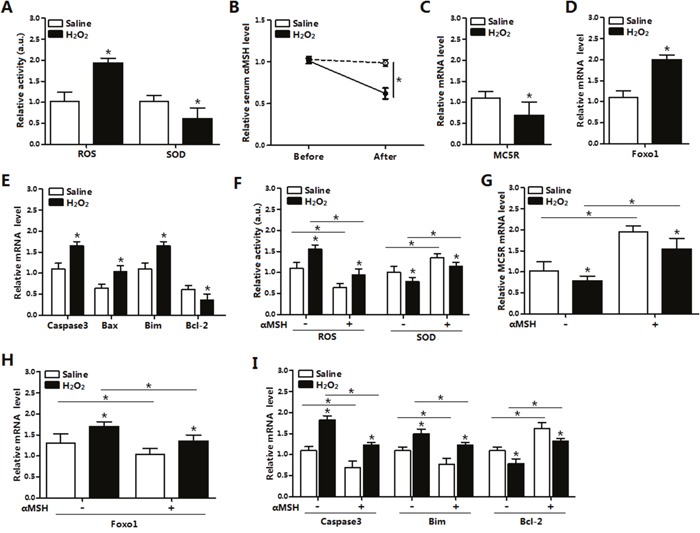
αMSH inhibited H2O2-induced oxidative stress, *Foxo1* expression and apoptosis in mice adipose tissue **(A)** Serum activity of ROS and SOD of mice with intraperitoneal injection of H_2_O_2_ (300 mg/kg/day) for 5 days or with saline injection (n=6). **(B)** Serum αMSH level before and after injection in both groups (n=6). **(C)** Relative mRNA levels of *MC5R* in the H_2_O_2_ and Control group (n=6). **(D)** Relative mRNA levels of *Foxo1* in the H_2_O_2_ and Control group (n=6). **(E)** Relative mRNA levels of *Caspase3, Bax, Bim* and *Bcl-2* in the H_2_O_2_ and Control groups (n=6). **(F)** Serum activity of ROS and SOD of mice with injection of αMSH, after injection of H_2_O_2_ or saline for 5 days (n=6). **(G)** Relative *MC5R* mRNA levels with injection of αMSH, after injection of H_2_O_2_ or saline for 5 days (n=6). **(H)** Relative *Foxo1* mRNA levels with injection of αMSH, after injection of H_2_O_2_ or saline for 5 days (n=6). **(I)** Relative *Caspase3, Bim, Bcl-2* mRNA levels with injection of αMSH, after injection of H_2_O_2_ or saline for 5 days (n=6). Values are means ± SD. vs. Control group, *p < 0.05.

Hydrogen Peroxide (H_2_O_2_) was injected intraperitonealy in mice for continuous 5 days and then mice were sacrificed. In mice inguinal tissue, ROS activity is found significantly increased and superoxide dismutase (SOD) activity is greatly decreased (*p* < 0.05, Figure 1A), which indicated the oxidative stress model on mice was established. Compared with resting status, the serum αMSH level and the *MC5R* mRNA level were decreased in the established oxidative stress status (*p* < 0.05, Figure 1B, 1C). While, *Foxo1* mRNA level was increased significantly in inguinal adipose tissue after H_2_O_2_ injection (Figure 1D). Moreover, the mRNA levels of pro-apoptotic genes, such as *Caspase3*, *Bax* and *Bim* were up-regulated, and the anti-apoptotic gene *Bcl-2* mRNA was down-regulated (*p* < 0.05, Figure 1E). In mice with 5 day H_2_O_2_ injection, αMSH was additionally injected for another 3 days. Opposite with H_2_O_2_ injection only, serum ROS activity was reduced, while the SOD activity and *MC5R* mRNA was increased (*p* < 0.05; Figure 1F, 1G) after the addition of αMSH. Figure 1H showed αMSH decreased *Foxo1* mRNA (*p* < 0.05). mRNA levels of *Caspase3* and *Bim* were also up-regulated (*p* < 0.05) under treatment of αMSH, while *Bcl-2* was down-regulated (*p* < 0.05; Figure 1I). Thus, we concluded that αMSH reduced H_2_O_2_-induced oxidative stress and apoptosis in adipose tissue of mice.

### ROS triggered apoptosis through causing oxidative stress in adipocytes

**Figure 2:**
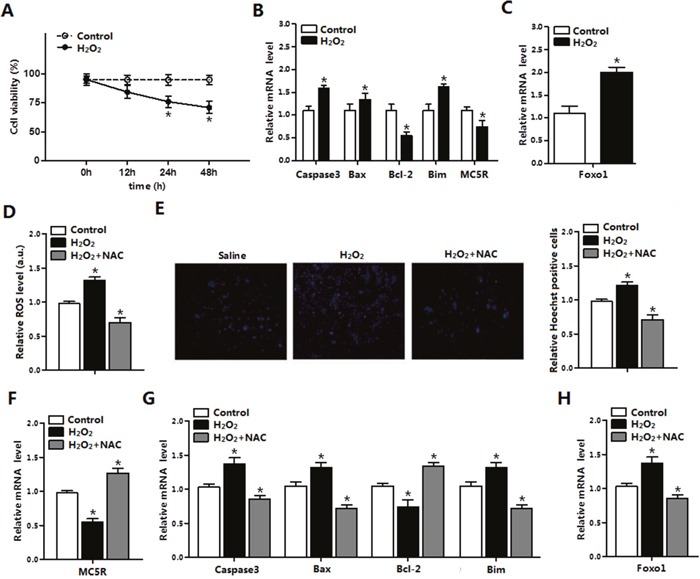
ROS triggered apoptosis through causing oxidative stress in adipocytes **(A)** Cell viability measurement in control group and H_2_O_2_ group after treatment with H_2_O_2_ for 12 h, 24 h, 48 h (n=3). **(B)** The relative mRNA levels of *Caspase3, Bax, Bcl-2, Bim* and *MC5R* in the H_2_O_2_ group and control group (n=3). **(C)** The relative mRNA levels of *Foxo*1 in control group and H_2_O_2_ group (n=3). **(D)** The relative ROS level of cells in the control group, the H_2_O_2_ group and the H_2_O_2_ and NAC co-treatment group (n=3). **(E)** The relative Hoechst Positive cells in each group; Hochest staining after H_2_O_2_ and NAC treatment in primary adipocytes (n=3). **(F)** The relative mRNA expression of *MC5R* in the control group, the H_2_O_2_ group and the H_2_O_2_ and NAC co-treatment group (n=3). **(G)** The relative mRNA expression of *Caspase3, Bax, Bcl-2* and *Bim* in the control group, the H_2_O_2_ group and the H_2_O_2_ and NAC co-treatment group (n=3). **(H)** The relative mRNA expression of *Foxo1* in the control group, the H_2_O_2_ group and the H_2_O_2_ and NAC co-treatment group (n=3). Values are means ± SD. vs. Control group, *p < 0.05.

To further investigate the role of ROS in adipocyte apoptosis, mature adipocytes were treated with H_2_O_2_ and saline was used as control. Results showed that H_2_O_2_ significantly decreased cell viability after 24 h and 48 h treatment (Figure 2A). The mRNA levels of *Caspase3, Bax*, and *Bim* were increased (*p* < 0.05), while *Bcl-2* and *MC5R* were decreased (*p* < 0.05) in H_2_O_2_ group (Figure 2B). The mRNA level of *Foxo1* was increased in H_2_O_2_ group (Figure 2C), which was consistent with the results *in vivo*. Then we used H_2_O_2_ and NAC co-treatment to determine how H_2_O_2_ affect adipocytes apoptosis. The ROS level and number of Hoechst positive cells were increased in H_2_O_2_ group. However, the oxidative stress inhibitor-NAC remarkably decreased the ROS level (*p* < 0.05) and the number of apoptosis cells (*p* < 0.05, Figure 2D, 2E). Moreover, mRNA expression of *Caspase3, Bax, Bim* and *MC5R* (Figure 2F) were all down-regulated (*p* < 0.05), while *Bcl-2* was increased (*p* < 0.05) in H_2_O_2_ and NAC co-treated group (Figure 2G). *Foxo1* was increased in the H_2_O_2_ group while decreased in the H_2_O_2_ and NAC co-treatment group significantly (Figure 2H). Thus, our results suggested that ROS enhanced apoptosis and the oxidative stress inhibitor-NAC reversed this phenomenon in mice adipocytes.

### αMSH inhibited oxidative stress and apoptosis via *MC5R* in adipocytes

**Figure 3:**
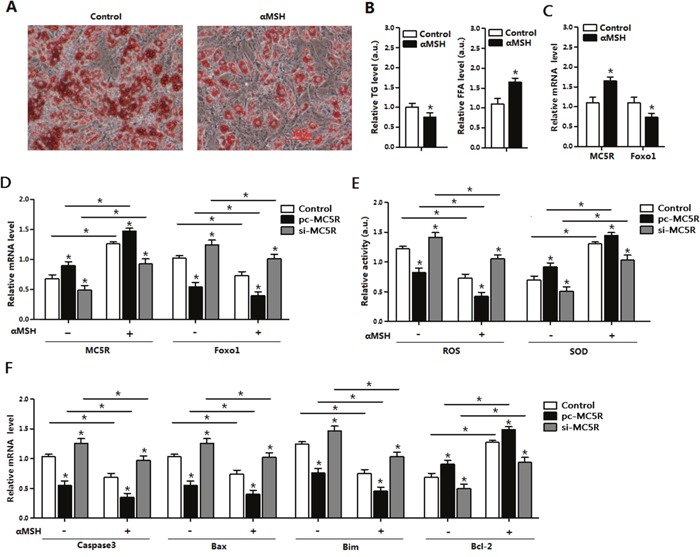
αMSH and *MC5R* inhibited oxidative stress, apoptosis and *Foxo1* expression in mice adipocytes **(A)** Images of adipocytes stained by Oil Red O staining after treatment of αMSH on differentiation until 6 day (n=6). **(B)** Relative TG level and FFA level after treatment of αMSH on differentiation until 6 day (n=6). **(C)** Relative mRNA levels of *MC5R* and *Foxo1* in the control group and αMSH treatment group (n=6). **(D)** The mRNA expression of *MC5R* and *Foxo1* after transfection with control vector, pc-*MC5R* and si-*MC5R* (n=6). **(E)** The relative activity of ROS and SOD after transfection with control vector, pc-*MC5R* and si-*MC5R* (n=6). **(F)** The mRNA expression of *Caspase3, Bax, Bim*, and *Bcl-2* after transfection with control vector, pc-*MC5R* and si-*MC5R* (n=6). pc-*MC5R*: *MC5R* overexpression vector; si-*MC5R*: *MC5R* shRNA vector; Control: pcDNA 3.1 vector. Values are means ± SD. vs. Control group, *p < 0.05.

Mature adipocytes were incubated with αMSH for 1h, then Oil Red O staining was processed. Results showed αMSH strongly promoted the lipolysis and FFA release in adipocytes (Figure 3A) and significantly reduced TG level (Figure 3B). Compared with non-treatment control group, the *MC5R* level and and the SOD activity were increased, while *Foxo1 and* ROS activity was reduced in cells treated with αMSH (Figure 3C-3E) which implicated the repressive role of αMSH in oxidative stress. To investigate the role of *MC5R* in this process, vectors were transfected into adipocytes to enhance or knock-down its expression, pcDNA3.1 empty vector was used as control. On the basis of αMSH treatment, when *MC5R* was over-expressed, the mRNA level of *Foxo1* and ROS activity were even lower and the SOD activity was much higher (*p* < 0.05; Figure 3D-3E). The enhanced trend also appeared in the mRNA level of *Capsase3, Bax Bim* and *Bcl-2* genes (Figure 3F). However, when *MC5R* was knocked down, the inhibitory function of αMSH in oxidative stress was correspondently blocked. These results demonstrated that the suppressive function of αMSH in oxidative stress and apoptosis is through targeting *MC5R* in mice adipocytes.

### αMSH inhibited ROS-induced apoptosis in adipocytes

**Figure 4:**
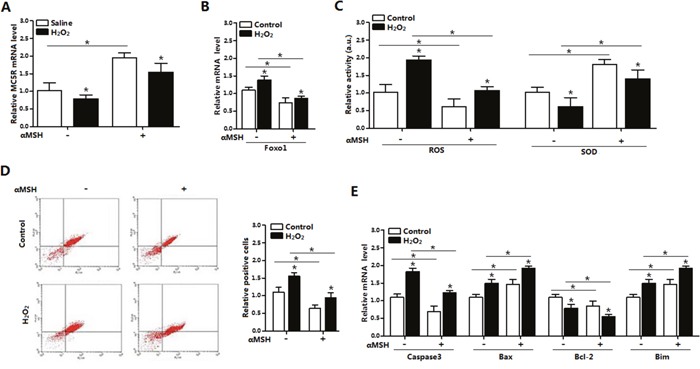
αMSH inhibited ROS-induced apoptosis and reduced *Foxo1* expression in mice adipocytes **(A)** The relative *MC5R* mRNA level of adipocytes with treatment of αMSH after incubated for 24 h in the presence of H_2_O_2_ (n=3). **(B)** The relative *Foxo1* mRNA level of adipocytes with treatment of αMSH after incubated for 24 h in the presence of H_2_O_2_ (n=3). **(C)** The relative activity of ROS and SOD in adipocytes treatment of αMSH after incubated for 24 h in the presence of H_2_O_2_ (n=3). **(D)** Annexin V-FITC/PI staining of apoptosis by flow cytometry analysis (n=3). **(E)** The relative mRNA level of *Caspase3, Bax, Bcl-2* and *Bim* of adipocytes with treatment of αMSH after incubated for 24 h in the presence of H_2_O_2_ (n=3). Values are means ± SD. vs. Control group, *p < 0.05.

We further examined the role of αMSH in the ROS-induced apoptosis. Mature adipocytes were pre-incubated with H_2_O_2_ first, significant increases were found in ROS activity and *Foxo1* mRNA, while great reductions were detected in SOD activity and *MC5R* mRNA, when compared with saline treatment (*p* < 0.05; Figure 4A-4C). By using Annexin V-FITC staining and flow cytometry, we found elevated number of apoptotic cells in H_2_O_2_ group, compared with saline group (*p* < 0.05; Figure 4D). In the apoptosis related genes, the mRNA level of *Caspase 3, Bax* and *Bim* were up-regulated, while *Bcl-2* are down-regulated after H_2_O_2_ treatment (*p* < 0.05, Figure 4E). However, adding of αMSH on the basis of H_2_O_2_ repressed all the apoptotic effects caused by H_2_O_2_. These data indicated that αMSH can inhibit ROS-induced apoptosis in adipocytes.

### Foxo1 enhanced ROS-induced apoptosis by positive transcriptional regulation of *Bim* and reverse the inhibitory function of αMSH and *MC5R* on oxidative stress

**Figure 5:**
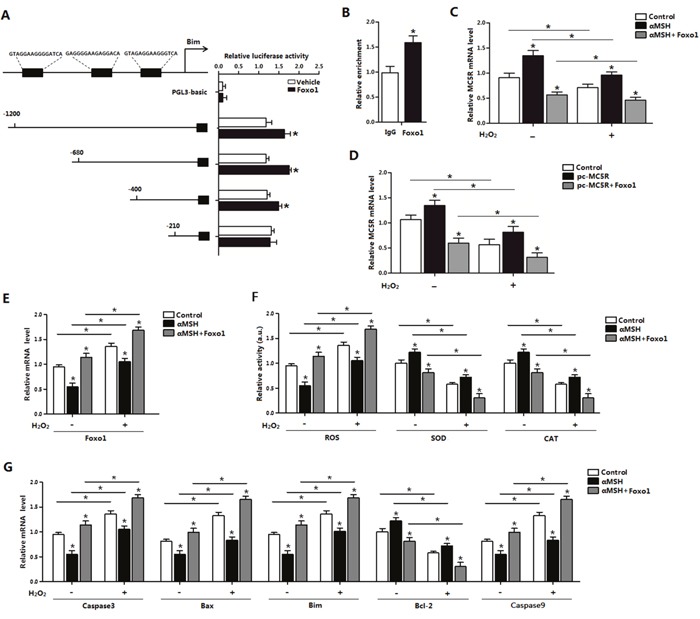
Effect of Foxo1 on *Bim* and αMSH in ROS-induced apoptosis **(A)** Fragments of *Bim* promoter fused to a luciferase reporter gene were co-transfected into cells together with PGL3-basic (control) or pc-*Foxo1* (n=3). Luciferase activity was corrected for Renilla luciferase activity and normalized to control activity (n=3). **(B)** ChIP analysis of Bim and Foxo1 in adipocytes (n=3). **(C)** Relative *MC5R* mRNA levels of adipocytes with treatment of αMSH and pc-*Foxo1* transfected for 48 h under oxidative stress induced by H_2_O_2_ (n=3). **(D)** Relative *MC5R* mRNA expression of adipocytes with pc-*MC5R* and pc-*Foxo1* transfected for 48 h under oxidative stress induced by H_2_O_2_ (n=3). **(E)** Relative *Foxo1* mRNA levels of adipocytes with treatment of αMSH and pc-*Foxo1* transfected for 48 h under oxidative stress induced by H_2_O_2_ (n=3). **(F)** Relative activities of ROS, SOD and CAT in cells with treatment of αMSH and pc-*Foxo1* transfected for 48 h under oxidative stress induced by H_2_O_2_ (n=3). **(G)** Relative mRNA expression of *Caspase3, Bax, Bim, Bcl-2* and *Caspase9* in adipocytes with pc-*MC5R* and pc-*Foxo1* transfected for 48 h under oxidative stress induced by H_2_O_2_ (n=3). Values are means ± SD. vs. Control group, *p < 0.05.

We next explored the role of Foxo1 in the process of αMSH inhibiting ROS-induced apoptosis. By using luciferase reporter assay, we identified the -400 - -210 promoter region of *Bim* was the binding site for Foxo1 (Figure 5A). The interaction between Foxo1 and *Bim* was confirmed by the ChIP analysis (Figure 5B). Moreover, to verify the effects of Foxo1 on αMSH and *MC5R*, we used pc-MC5R and pc-Foxo1 plasmid to transfer cells. Results indicated that forced expression of *Foxo1* reversed the enhanced effection of αMSH and pc-MC5R on *MC5R* expression (*p* < 0.05; Figure 5C, 5D). We further detected the level of ROS, SOD and CAT as well as the marker genes of apoptosis after forced expression of *Foxo1*. Enhanced expression of Foxo1 not only repressed, but reversed the effect of αMSH, which are shown in Figure 4, in all these aspects. The elevated SOD, CAT activity and *Bcl-2* expression lead by αMSH was shown to be decreased in αMSH and Foxo1 co-treated group, regardless of the present of H_2_O_2_ treatment; while the reduced *Foxo1* expression, ROS activity and mRNA level of pro-apotosis factors *Caspase3*, *Bax*, *Bim* and *Caspase 9*, which brought by αMSH, were shown increased after adding Foxo1 (*p* < 0.05; Figure 5E-5G). Thus, our data clearly showed that Foxo1 is a positive transcriptional factor of *Bim* and its enhancement function in apoptosis is so strong that can even reverse the the inhibitory effect on ROS-induced apoptosis by either αMSH or *MC5R* treatment.

### αMSH inhibited apoptosis through reducing *Bim* and *Foxo1* expression

**Figure 6:**
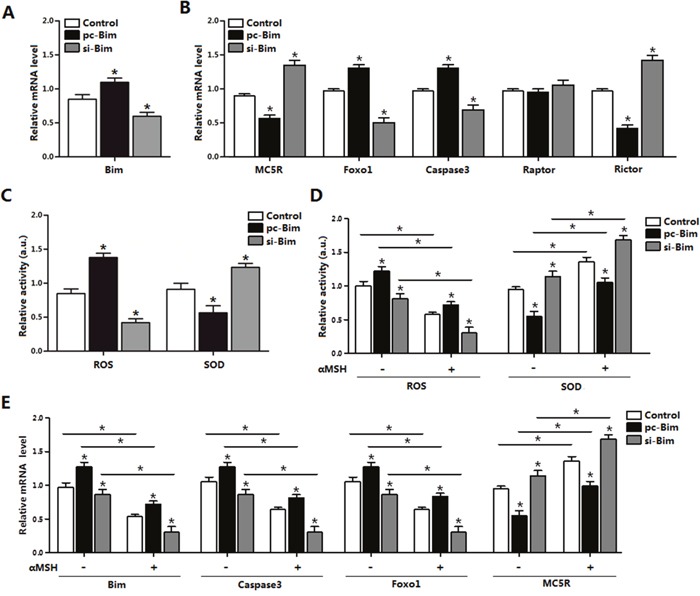
αMSH inhibited apoptosis through reducing *Bim* and *Foxo1* expression **(A)** *Bim* transfection efficiency detection in Control group, pc-*Bim* group and sh-*Bim* group after 48 h transfection (n=3). **(B)** Relative mRNA level of *MC5R, Foxo1, Caspase3, Raptor* and *Rictor* in Control group, pc-*Bim* group and sh-*Bim* group after 48h transfection (n=3). **(C)** Relative activity of ROS and SOD in Control group, pc-*Bim* group and sh-*Bim* group after 48 h transfection (n=3). **(D)** Relative activities of ROS and SOD in Control group, pc-*Bim* group and sh-*Bim* group after 48 h transfection under treatment of αMSH (n=3). **(E)** Relative mRNA levels of *Bim, Caspase3, Foxo1* and *MC5R* in Control group, pc-*Bim* group and sh-*Bim* group after 48 h transfection under treatment of αMSH (n=3). pc-*Bim*: *Bim* overexpression vector; si-*Bim*: *Bim* shRNA vector; Control: pcDNA 3.1 vector. Values are means ± SD. vs. Control group, *p < 0.05.

To further define the relationship between *Bim* and αMSH, we first transfected cells with *Bim* overexpression or interference vector. The transfection efficiency of *Bim* was determined (Figure 6A). As expected, *Bim* was 120% greater in the *Bim* overexpression group compared with control group, while decreased 45% after *Bim* was stable knocked down (Figure 6A). Forced expression of *Bim* increased the mRNA levels of *Foxo1, Caspase3*, while decreased *MC5R* (*p* < 0.05; Figure 6B). Interestingly, *Bim* increased the expression of *Rictor*, a component of mTORC2 (*p* < 0.05), while the mTORC1 component *Raptor* was not changed (Figure 6B). Overexpressed of *Bim* increased the cells oxidative stress, since the ROS activity increased and SOD activity decreased (Figure 6C, 6D). Over-expression of *Bim* can repress the effect of αMSH on mRNA level of *Bim*, *Caspase3*, Foxo1 and MC5R, vice versa. (*p* < 0.05; Figure 6E). From these results, we can declared that Bim itself can triger oxidative stress and act as a key regulator in αMSH inhibited apoptosis.

### Rictor/mTORC2 signaling pathway was activated during αMSH inhibiting adipocyte apoptosis and *Foxo1* expression

**Figure 7:**
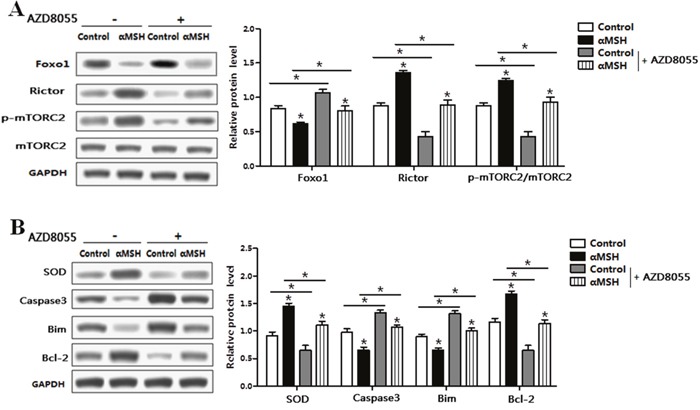
αMSH inhibited adipocyte apoptosis via Rictor/mTORC2 signaling pathway Adipocytes were pretreated with αMSH or control, and then treated with AZD8055 or not. **(A)** Representative immunoblots and densitometric quantification for Foxo1, Rictor, p-mTORC2 and total mTORC2 (n=6). **(B)** Representative immunoblots and densitometric quantification for SOD, Caspase3, Bim and Bcl-2 (n=6). Values are means ± SD. vs. Control group, *p < 0.05.

To further characterize the underlying mechanisms of αMSH on adipocyte apoptosis, we determined the mTORC2 pathway signal using the specific inhibitor AZD8055. The protein level of Rictor and the phosphorylation of mTORC2 were both elevated when protein level of Foxo1 was decreased by αMSH (Figure 7A). Conversely, AZD8055 treatment decreased mTORC2 phosphorylation level and protein expression of Rictor, while increased the protein level of Foxo1 (Figure 7A). We further detected the protein levels of apoptotic markers and oxidative stress, αMSH enhanced the protein levels of SOD and Bcl-2, while reduced protein levels of Caspase 3 and Bim (*p* < 0.05; Figure 7B). At the same time, AZD8055 decreased the protein level of SOD and Bcl-2, while increased protein levels of Caspase 3 and Bim (Figure 7B). These results implied that Rictor/mTORC2 signaling pathway was activated during αMSH inhibiting adipocyte apoptosis.

## DISCUSSION

αMSH rescues neurons from apoptosis induced by other insults, including traumatic brain injury, cerebral ischemia and hippocampal excitotoxicity [22]. Pretreatment of αMSH significantly inhibits dexamethasone-induced apoptosis and necrosis in both osteoblastic-like MC-3T3-E1 cells and primary murine osteoblasts [23]. Apoptosis can be prevented by αMSH in retinal vascular cells, neuroretina of early diabetic retinas [24] and M17 neuroblastoma cells through reducing the level of cleaved Caspase-3 and attenuating Cytochrome C release [25]. αMSH is also reported to suppresses the oxidative stress induced by ultraviolet radiation in skin keratinocytes and melanocytes [26–28]. In this study, we established oxidative stress model with H_2_O_2_, and found enhanced apoptosis by H_2_O_2_ in both adipose tissue and primary adipocyte of mice. Moreover, we found both αMSH and *MC5R* expression decreased along with the ROS-induced apoptosis in mice adipocyte. By treatment with αMSH and forced expression of *MC5R*, we found *Caspase3*, *Bax* and *Bim* were decreased and *Bcl-2* was increased, which indicated the anti-apoptosis role of αMSH. These results demonstrated that αMSH could reverse apoptosis induced by ROS in adipocytes.

*Foxo* subfamily is associated with the induction of apoptosis in various cell types [29–32]. In addition, Foxo3a inhibits ROS-induced apoptosis in undifferentiated 3T3-L1 cells via the expression of ROS-scavenging enzymes [33]. We found *Foxo1* expression was increased in ROS-induced adipocyte apoptosis. Moreover, αMSH decreased the expression of *Foxo1* in mice adipose tissue and adipocytes. Then we hypothesized the opposite relationship of αMSH and *Foxo1* in the ROS-induced apoptosis. Our results indicated αMSH inhibits ROS-induced apoptosis by suppressing *Foxo1*. It has been reported that activated Foxo proteins potentiate pro-apoptotic protein Bim expression [34–37]. Bim is essential for the death of neurocyte, epithelium cardiomyocyte and oncocytes [38–40]. Sun et al. report that Foxo1 enhances the transcription of its pro-apoptotic target *Bim*, and Foxo1-Bim mediates caffeine-induced regression of glioma growth by activating cell apoptosis [41]. In our study, we also confirmed Foxo1 is a positive transcription factor of *Bim* in adipocytes. The results also showed the forced expression of *Foxo1* reversed the inhibitory effect of αMSH on ROS-induced apoptosis.

mTOR signaling is inhibited by Foxo1 in mammalian skeletal muscle [18]. Moreover, concurrent inhibition of PI3K and mTORC1/mTORC2 overcomes resistance to rapamycin induced apoptosis in mantle cell lymphoma [42]. In our results, the increased level of p-mTORC2/mTORC2 was found during αMSH treatment. Oxidative stress and apoptosis factors were also reduced by αMSH. These results declared for the first time that Rictor/mTORC2 signal was activated during the repression of αMSH in ROS induced adipocyte apoptosis.

**Figure 8:**
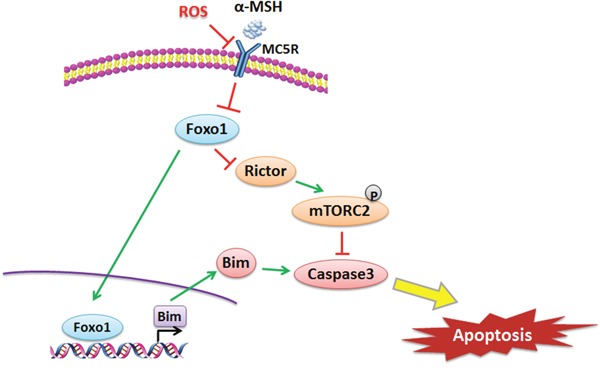
αMSH inhibited apoptosis induced by ROS via Foxo1/mTORC2 signal αMSH reduced *Foxo1* through MC5R, then down-regulated *Bim* and up-regulated *Rictor*, which inhibited apoptosis via mTORC2. Foxo1 is a transcriptional activator of *Bim* and can aggravate ROS-induced apoptosis by binding to *Bim* promoter region in adipocytes.

In conclusion, our results demonstrated that αMSH inhibited ROS-induced apoptosis is through reducing Foxo1 and activating Rictor/mTORC2 signal in mice adipocytes. (summarized in Figure 8). Moreover, we proved that Foxo1 is a novel transcriptional activator of *Bim* and can aggravate ROS-induced apoptosis by binding to *Bim* promoter region in adipocytes. These findings shed new light on the study of molecular mechanism of metabolic disease, made Foxo1 as a potential target for medicine development.

## MATERIALS AND METHODS

### Animal experiment

Six-week-old Kunming male mice were purchased from the Laboratory Animal Center of the Fourth Military Medical University (Xi’an, Shaanxi, China). All mice were carried out in accordance with applicable guidelines and regulations approved by the Animal Ethics Committee of Northwest A&F University. Mice were allowed ad libitum access to water and standard chow laboratory diet (Animal Center of the Fourth Military Medical University). Animal room was maintained under controlled conditions of temperature at 25°C ± 1 °C, humidity at 55 ± 5%, and a 12 h light/12-dark cycle. Body weight was recorded once a week. H_2_O_2_ (300 mg/kg/day) was intraperitoneal injected into eight-week-old mice for 5 days. αMSH (1 mg/kg/day) or saline was then subcutaneous injected for 3 days before mice were euthanized. Tissues and blood were harvested and serum triglyceride (TG) level was measured using the Infinity Triglyceride kit (Sigma, St. Louis, USA), serum αMSH level was measured using commercial ELISA kit (R&D Systems, USA).

### Primary adipocytes culture and cell viability assay

Inguinal white adipose tissues were harvested, visible fibers and blood vessels were removed and the adipose tissue was washed three times with PBS buffer containing 200U/mL penicillin (Sigma, St. Louis, USA) and 200U/mL streptomycin (Sigma, St. Louis, USA). The adipocyte culture was performed as previously described [43]. Oil Red O staining was conducted to measure lipid droplets as previously described [44]. Cell viability was measured as previously described [44].

### Drug treatments and transfection of adipocytes with plasmids

H_2_O_2_ (Sigma, St. Louis, MO, USA) working solution (0.5mM) were prepared to treat cells for 24 h. The αMSH (300 nM, Sigma, St. Louis, MO, USA) was mixed to treat mature adipocytes for 1 h. To study the molecular mechanism of signaling pathways, on the 6th day of cell differentiation, αMSH treatment group and control group were treated with 1 μM mTORC2-specific inhibitor AZD8055 for 48 h.

Forced expression plasmid vectors of *MC5R* (pc-*MC5R*), *Foxo1* (pc-Foxo1) and *Bim* (pc-*Bim*) were kept in our lab; and the control plasmid vector was pcDNA3.1-vector. shRNA sequence against *MC5R* (si-*MC5R*) and *Bim* (si-*Bim*) were contrived and synthesized by Genepharma Company (Shanghai, China) using pGPU6/Neo siRNA expression vector. The cell transfection was performed as previously described [45].

### Measurement of oxidative stress and adipocyte apoptosis assay

For intracellular ROS detection, cell-permeable non fluorescent probe 2’, 7’-dichlorofluorescin diacetate (DCFH-DA) (Beyotime, China) dying assay was used. The dye loading was performed by incubating the adipocytes with 10μM DCFH-DA at 37°C for 60min. The production of ROS was examined using a spectrophotometer by measuring the fluorescence intensity of DCF at an excitation wavelength of 488 nm and emission wavelength of 525 nm. Superoxide dismutase (SOD) activity and catalase (CAT) activity measurements were performed using the commercially available kits from Beyotime Co. (Nanjing, China) according to the instruction for authors.

Nuclear morphology change was observed by using Hoechst 33258 fluorochrome stain. Cells were washed three times in PBS buffer and then were fixed in 4.0% paraformaldehyde. The fixed cells were then washed with PBS three times and stained with Hoechst 33258 (5 μM) for 15 min, washed with PBS three times again. Apoptosis associated nuclear alterations were examined under UV filter using Olympus (TH-4-200, USA) microscope with fluorescence attachment. Annexin V-FITC Staining was further used to measure cell apoptosis. Cells were washed two times in PBS buffer. Add 5uL Annexin V fluorescein isothiocynate (FITC) and 5uL PI Staining Solution, then incubated for 10 min. Acquisition of stained cells was done with flowcytometer (Beckman, USA) and data analysis was performed with Diva software (BD Biosciences, American).

### Luciferase report assay and chromatin immunoprecipitation (ChIP) assays

Four fragments containing Bim-5’ sequences were from -1200 to -210 relative to the transcription initiation site was sub-cloned into pGL3-basic vector (Takara, China), respectly. HEK293T cells were cultured in 24-well plates and co-transfected with Bim promoter plasmid and pc-Foxo1 plasmid or pGL3-basic plasmid (control reporter). Cells were harvested 48 h after transfection, and detected using the Dual-Luciferase Reporter assay system (Promega, USA). And luciferase activity was divided by all luciferase assay experiments were performed three times at least and each conducted in triplicate.

Chromatin immunoprecipitation (ChIP) assay was performed by using a ChIP assay kit (Abcam, Cambridge, UK) according to the manufacturer's protocol. DNA-protein crosslinking complexes were collected, and purified DNA was subjected to qPCR with SYBR green fluorescent dye (Invitrogen, Californian, USA).

### Real-time quantitative PCR analysis and western blot analysis

Total RNA from adipose tissues or adipocytes were extracted with TRIpure Reagent kit (Takara, Dalian, China) and 400 ng of total RNA was reverse transcribed using the M-MLV reverse transcriptase kit (Takara, China). Primers for *MC5R*, *Foxo1*, *Caspase3*, *Caspase9*, *Bax*, *Bim*, *Bcl-2*, *Raptor* and *Rictor* were synthesized by Shanghai Sangon Ltd (Shanghai, China). Quantitative PCR was performed in 25 μL reactions containing specific primers and SYBR Premix EX Taq (Takara, Dalian, China). The levels of mRNAs were normalized to *β-actin*. The expression of genes were analyzed by method of 2^-ΔΔCt^.

Cells were lysed in RIPA buffer for 40 min at 4°C. Removing insoluble material by centrifugation at 12,000 × g for 15 min at 4°C, and the supernatants were used to assay protein levels. Protein samples (50 μg) were separated by electrophoresis on 12% and 5% SDS-PAGE gels using slab gel apparatus and then transferred to PVDF nitrocellulose membranes (Millipore, USA) blocked with 5% Skim Milk Powder/Tween 20/TBST at room temperature for 2 h. Antibodies against Foxo1, Rictor, p-mTORC2, mTORC2, SOD, Caspase3, Bim, Bcl-2, GAPDH and mTORC2 special inhibitor AZD8055 were from Abcam (Cambrige, England). Membranes were incubated with primary antibodies at 4°C overnight and then incubated with the appropriate HRP-conjugated secondary antibodies (Boaoshen, China) for 2 h at room temperature. Proteins were visualized using chemiluminescent peroxidase substrate (Millipore), and then the blots were quantified using ChemiDoc XRS system (Bio-Rad) and Quantitative analysis of immune-blotted bands was performed using Quality One software (Bio-Rad).

### Statistical analysis

Statistical calculations were performed with SAS v8.0 (SAS Institute, Cary, NC). Statistical significance was determined using the one-way ANOVA test. Comparisons among individual means were made by Fisher's least significant difference (LSD) post hoc test after ANOVA. Data are presented as mean ± SD; *p* < 0.05 was considered to be significant.
